# Avian community structure and habitat use of *Polylepis* forests along an elevation gradient

**DOI:** 10.7717/peerj.3220

**Published:** 2017-04-27

**Authors:** C. Steven Sevillano-Ríos, Amanda D. Rodewald

**Affiliations:** Department of Natural Resources, Cornell University, Ithaca, NY, United States of America; Cornell Lab of Ornithology, Conservation Science Lab, Ithaca, United States of America

**Keywords:** Andean birds, Climate change, Conservation, Endemics, Threatened species, *Polylepis* birds, Montane

## Abstract

**Background:**

As one of the highest forest ecosystems in the world, *Polylepis* forests are recognized both as center of endemism and diversity along the Andes and as an ecosystem under serious threat from habitat loss, fragmentation, and climate change due to human activities. Effective conservation efforts are limited, in part, by our poor understanding of the ecology and habitat needs of the ecosystem’s flora and fauna.

**Methods:**

In 2014–2015, we studied bird communities and 19 associated local and landscape attributes within five forested glacial valleys within the Cordillera Blanca and Huascaran National Park, Peru. We surveyed birds during the dry (May–August) and wet (January–April) seasons at 130 points distributed along an elevational gradient (3,300–4,700 m) and analyzed our data using Canonical Correspondence Analysis (CCA).

**Results:**

We associated a total of 50 species of birds, including 13 species of high conservation concern, with four basic habitat types: (1) *Polylepis sericea* forests at low elevations, (2) *P. weberbaueri* forests at high elevations, (3) Puna grassland and (4) shrublands. Four species of conservation priority (*e.g., Microspingus alticola*) were strongly associated with large forest patches (∼10-ha) of *P. sericea* at lower elevations (<3,800 m), whereas another four (*e.g., Anairetes alpinus*) were associated with less disturbed forests of *P. weberbaueri* at higher elevations (>4,200 m).

**Discussion:**

Results suggest two key strategies form the cornerstones of conservation efforts: (a) protect large remnant (>10-ha) *P. sericea* forests at lower elevations and (b) maintain all relicts of *P. weberbaueri*, irrespective of size, at high elevations (>4,200 m).

## Introduction

Tropical mountains are well known to support impressively high species diversity and endemism (Körner, Nakhutsrishvili & Spehn, 2006; Spehn et al., 2012), and the tropical Andes, in particular, stand out as a biodiversity hotspot (Myers et al., 2000; Antonelli & San Martín, 2011; Jenkins, Pimm & Joppa, 2013). One unique Andean ecosystem, nestled in the humid and dry Puna along the Andes, is the *Polylepis* forest (Simpson, 1979; Simpson, 1986; Kessler, 2006). Considered as one of the world’s highest elevation forests (Gareca et al., 2010), it represents a center of avian diversity (Fjeldså et al., 1996; Fjeldså, 2002) and endemism (Fjeldså, Lambin & Mertens, 1999; Fjeldså, 1993), with several birds restricted to this specific ecosystem (Fjeldså & Kessler, 2004; Gareca et al., 2010; Lloyd, 2008a; Lloyd, 2008b; Lloyd, 2008c; Lloyd & Marsden, 2008). According to Fjeldså (2002), 214 bird species use *Polylepis* forest along the entire range of the Andes, 51 of which are strongly associated to *Polylepis* and 14 that are highly specialized. Unfortunately, *Polylepis* forests continue to be threatened by habitat loss, fragmentation, and degradation (Renison, Hensen & Cingolani, 2004; Jameson & Ramsay, 2007; WCMC-IUCN, 1998) and face future threats from climate change (Şekercioğlu, Primack & Wormworth, 2012).

The Red List of Threatened Species of the International Union for Conservation of Nature (IUCN Red List) recognizes 23 bird species (1 CR, 6 EN, 7 VU and 9 NT) associated with *Polylepis* forest as globally threatened (Birdlife International, 2016). These species are thought to be particularly sensitive to human activities and habitat degradation, given their limited dispersal abilities (Lloyd & Marsden, 2011), high degrees of ecological specialization (Fjeldså, 1993; Servat, 2006), and small population sizes (Lloyd, 2008a). The Royal Cinclodes (*Cinclodes aricomae*) for example, is critically endangered (CR) with an estimated population of 250 individuals restricted to the *Polylepis* woodlands of southeast Peru (Cuzco, Apurimac, Ayacucho, and Junin) (Birdlife International, 2016; Aucca et al., 2015). Other endangered species restricted to *Polylepis* woodlands include the Ash-breasted Tit-tyrant (*Anairetes alpinus*), the Plain-tailed Warbling-finch (*Microspingus alticola*) and the White-browed Tit-spinetail (*Leptasthenura xenothorax*) (Lloyd, 2008a).

Although several studies have described bird communities associated with *Polylepis* woodlands in general terms (Fjeldså, 1987; Frimer & Nielsen, 1989; Fjeldså, 1992; Fjeldså & Kessler, 2004), few have identified specific habitat characteristics associated with different species (Herzog, Soria & Matthysen, 2003; Matthysen, Collet & Cahill, 2008; Lloyd, 2008a; Lloyd & Marsden, 2008; Tinoco et al., 2013). Even fewer have systematically surveyed species across the entire elevation gradient covered by the *Polylepis* ecosystem (Kessler et al., 2001). Recent studies show that many *Polylepis* specialist birds are closely associated both with the physical characteristics of the landscape (e.g., patch size, connectivity) (Lloyd & Marsden, 2008; Tinoco et al., 2013) and local habitat attributes (Lloyd, 2008c). Because *Polylepis* forests vary widely in plant structure, composition and abiotic conditions throughout their elevational and latitudinal ranges (Fjeldså, 2002; Kessler, 2006), we expect that there may be further specialization even within the *Polylepis* bird community. In other words, some species may be restricted to certain attributes of *Polylepis* habitats, or even associated with particular *Polylepis* species. For example, many species are likely to occupy only subsets of the elevational ranges of several *Polylepis* trees and shrubs, which are highly variable, and extend from as low as 900 m in the case of *P. australis* (Márcora et al., 2008) to as high as 5,200 m in the case of *P. tarapacana* (Troll, 1973; Simpson, 1979; Kessler, 2005). Thus, for *Polylepis* ecosystems, as with many globally-threatened ecosystems around the world, effective conservation requires study of fine-scale distributions patterns, species-habitat associations, and ecological relationships among species. This understanding is particularly important in cases where limited resources must be optimally allocated to ensure meaningful outcomes.

In this study of *Polylepis* ecosystems, we (1) describe shifts in the floristics and structure of *Polylepis* forests along an elevational gradient, (2) examine how bird communities changed with elevation, habitat attributes, and landscape characteristics, and (3) identify specific habitat and landscape attributes associated with threatened and endemic birds. We focused on the Cordillera Blanca of Peru because it contains some of the best remnant *Polylepis* forests and, therefore, can provide important insights about anthropogenic stressors to these sensitive ecosystems. A deeper understanding of bird-habitat relationships along the elevational gradient will provide essential information to guide conservation of specialized *Polylepis* communities and endangered species, which are subject to the dual threats of human activities and climate change (Şekercioğlu, Primack & Wormworth, 2012).

## Methods

### Study area

We conducted the research in Cordillera Blanca, the highest tropical mountain range in the world, located in Ancash Department in Peru (9°06′19″S, 77°36′21″W) (Fig. S1). Study sites were located within Huascaran National Park and Huascaran Biosphere Reserve, both protected since 1975 and declared a world heritage site by UNESCO in 1985 (SERNANPE, 2010). The complex topography of the study area includes 44 deep glacial valleys spanning extensive elevational gradients that, in only a few kilometers, ascend from 2,400 m to mountains reaching 5,000 m to 6,768 m at the peak of Huascaran, the world’s highest tropical mountain (Byers, 2000). Each valley included several patches of *Polylepis* forest surrounded by a matrix of bushes, grasslands, wetlands, lagoons and other plant communities. These forests represent the largest extents of protected *Polylepis* woodland in the world (Zutta, 2009; Zutta et al., 2012). Mean annual rainfall is ∼844 mm and is most plentiful at high elevations (Schauwecker et al., 2014). There is also a strong seasonality, with the year partitioned into dry (May to August) and wet (September through April) seasons, with precipitation peaking during January through March (∼130 mm per month). Mean annual temperature is 13.5 °C, but daily temperatures can plummet to −15 °C at night and soar to 23 °C at noon during the dry season.

We select five glacial valleys to study on the Pacific slope based on accessibility, the presence of broad elevational gradients, and spatial distribution along the Cordillera Blanca. Three parallel valleys ranging from 3,300 m to 4,700 m were located in the north of Cordillera Blanca (Parón, Llanganuco and Ulta), and two valleys (Llaca and Rajucolta) were located more centrally within the Cordillera, covering an elevational gradient from 3,800 m to 4,700 m. We collected the data from mid-May to mid-August 2014, corresponding to the dry season, and from mid-January to mid-April 2015, corresponding to the wet season. We surveyed birds and vegetation in the vicinity of 130 sampling points located along five glacial valleys (see below for more details).

### Habitat surveys

During each season, our field teams measured 19 habitat and landscape variables within 130 circulars plots with a 10-m radius (a 50-m radius for one variable), centered on each point and divided into four quadrants by the intersection of North–South and East–West axes at the plot center. We estimated the percentage of mosses, Puna grass, rocks and bare ground in each quadrant and later averaged for use in analysis. In addition, we measure the height and diameter at breast height (DBH) of the nearest tree (woody vegetation with individual main stems > 10 cm DBH) within each quadrant and the tree was identified to the species level. We measured and averaged the DBH of all stems when multi-stemmed trees were encountered. Using the collected data, we calculated the biomass using the allometric equation (Eq. 1) developed for *Polylepis* trees by Espinoza & Quispe (2005) after their study in HNP.

(1) \begin{eqnarray*}\mathrm{Biomass}=0.0694\ast DB{H}^{2.35996}.\end{eqnarray*}

We counted the total number of trees > 10 cm DBH by quadrant, and the number of shrubs (multi-stemmed woody vegetation ≤ 10 cm DBH; typically, *Lupinus*, *Senecio*, *Berberis*, *Baccharis*, *Gynoxys* and small *Polylepis*), to calculate tree and shrub density in the circular plot, adjusted for an area of 100 m^2^. We included a zero in the average for DBH or three height if there were trees in some of the quadrants but not in all. As an indicator of vertical forest structure, we also measured the canopy depth at each quadrant, defined as canopy height minus the height of the canopy base, and groundcover height (groundcover: vegetation ≤ 50 cm). We used the mean of these variables for the subsequent analysis. We used a spherical densiometer to estimate canopy cover at the center of every point. For the landscape measurements, we estimated the percentage of forest within a 50-m circular plot, the patch size of forest in ha (points outside forest were 0 hectares), and the distance from the point center to the nearest forest edge (positive values indicated inside the forest and negative outside). We calculated all metrics using Quantum GIS Geographic Information System (QGIS Development Team, 2009) and the OpenLayers Plugin 1.3.6 based on CNES/Astrium satellite images from Google Earth 2015 with 1-m resolution.

### Bird surveys

**Figure 1:**
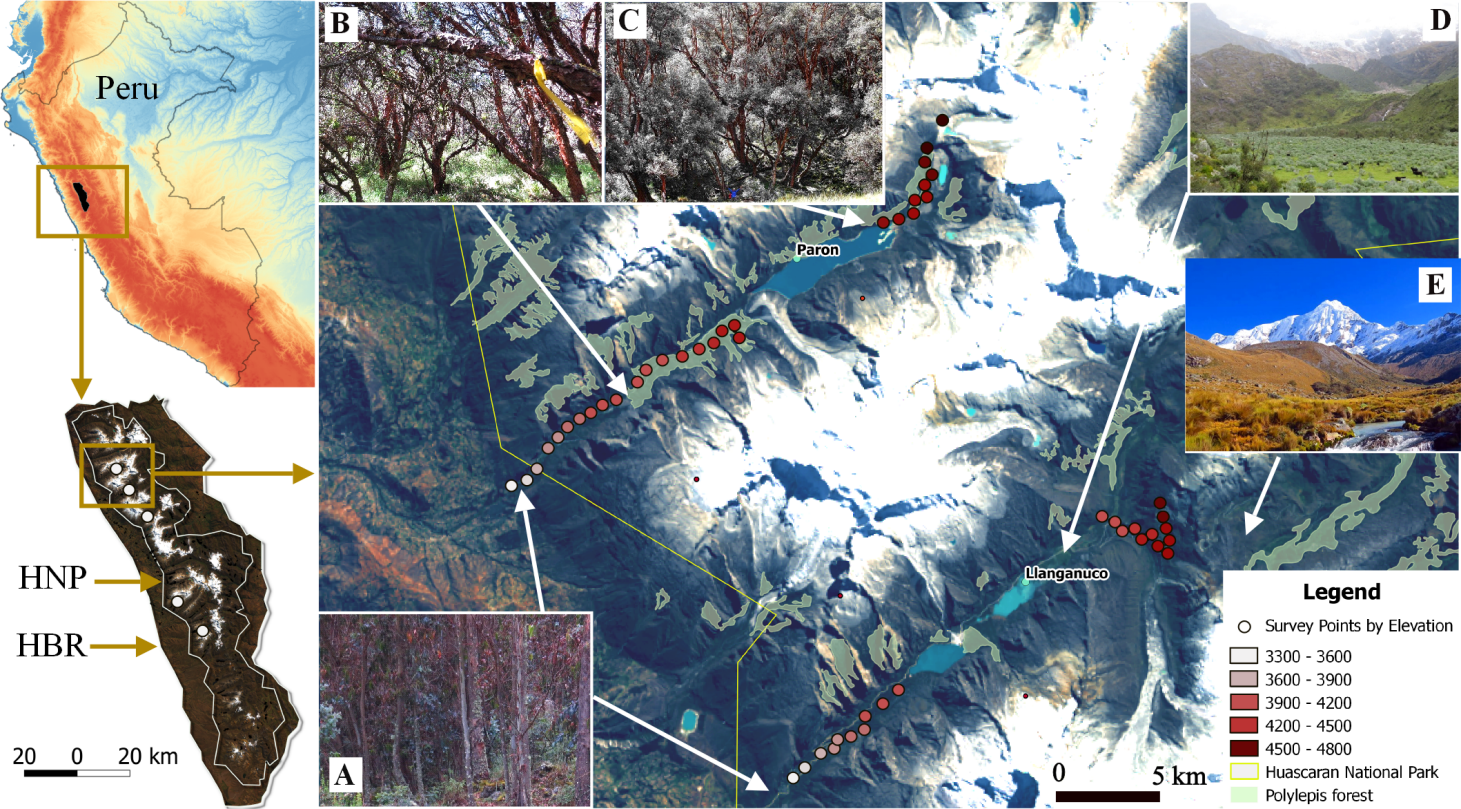
Study sites and vegetation communities located along an elevational gradient on Huascaran Biosphere Reserve (HBR) and Huascaran National Park (HNP)—Peru. On each of the five study sites (white dots on the bottom left map), Eucalyptus forest are usually at valley glacial entrances (A), *Polylepis sericea* is the dominant species at lower and middle elevations (B), while *P. weberbaueri* is at higher elevations (C). The matrix is mostly dominated by either by shrubby areas of *Gynoxys/Budleja/Baccharis/Lupinus* species (D) or Puna grassland areas dominated by *Stipa ichu* (E).

We used a robust sampling design for multiple species to survey the bird community (Jolly, 1965; Kendall, Nichols & Hines, 1997; Kendall, 2001). For each of the three large glacial valleys, we established three transects that included ten survey points stratified across habitats and along the gradient, whereas for the smaller valleys, we selected two transects of ten points on each. Each transect extended approximately 400 m along the elevation gradient within each glacial valley and did not overlap with other transects. Points were separated by >150 m (mean: 190 m, SD: 74.15, min–max: 150 m–480 m), haphazardly placed to ensure representation of the full range of habitat types in a valley and, stratified by elevation so as to span the entire elevational gradient of each valley (3,300 m–4,700 m). In total, we established 130 points along the five glacial valleys. In total, we located 130 points along the five glacial valleys. We do not detect spatial correlation on the variogram analysis with respect to species richness by each season (sill: 0.08 & 0.021, range: 0, nugget: 0.05 & 0.02 for the dry and wet season respectively) (Fig. S2). We recorded UTM eastings and northings coordinates and elevation (±10 m) at every point, and adjusted with a Digital Elevation Model (DEM) of 10 m resolution provided by the administration of Huascaran National Park (TerraSAR-X-Elevation10). We located a total of 30 points in each of the three larger valleys of Parón, Llanganuco and Ulta and 20 points in the smaller valleys of Llaca and Rajucolta. We located a total of 70 points inside woodlands dominated by *Polylepis* trees, 46 in areas dominated by shrubs and short-statured trees, such as *Gynoxys/Buddleja*, 6 within *Eucalyptus* forest, and 8 in Puna grassland (Fig. 1).

We surveyed all 130 points for 3 consecutive days during the dry and again in the wet season. During the 3-day sampling period, we visit each point three times during the dry season by a single observer, and five times during the wet season by two observers. At each point, the observer recorded all birds seen or heard within 50 m over a 10-min period. Surveys were conducted from sunrise (∼0500–0600 h) to ∼1200 h, and we reversed the order of surveys each visit to avoid bias related to bird activity, time of day, and/or observer experience (Lloyd, 2008a; Lloyd, 2008b). For each bird detection, we recorded time, species, number of individuals, linear distance from the point count center, and habitat type. We only counted once when individuals were detected multiple times. The Cornell Lab of Ornithology and the Institutional Animal Care and Use Committee (IACUC) of Cornell University provided full approval for this observational research. The entire field study was approved by Servicio Nacional de Areas Naturales Protegidas del Peru (SERNANPE), under the Resolution PNH-N. 014–2014.

### Data analysis

Because habitat variables did not differ significantly between seasons after we performed a paired *t* Wilcoxon test, we used the mean of the two seasons in all analyses. We tested for normality and independence of the habitat variables using Shapiro–Wilk W test (*p* < 0.01) and Spearman’s D correlation test (*r* < 0.75), respectively. We compared habitat attributes among the 5 valleys using the non-parametric Kruskal-Wallis test. We examined changes in habitat along the elevation gradient using a non-metric multidimentional scaling (NMDS) and Bray-Curtis similarity index on Past 3.08 (Hammer, Harper & Ryan, 2001).

We examined changes in forest composition (i.e., *Polylepis* spp.) with elevation using an occupancy model based on detection/no-detection data with elevation as the single covariate in program MARK (White & Burnham, 1999). Although the MARK occupancy models have been primarily applied to animals, they also can be used for plants and other sessile organism (Royle et al., 2005; Cheng et al., 2013; Kellner & Swihart, 2014). We tested for goodness of fit of including elevation on the model through a likelihood ratio test performed in program MARK. Because we expected that trees > 10 cm DBH would be easily detected in a small plot wherever they are present, we also fit a fixed occupancy logistic regression to the data (i.e., detection probability of one) and compared the AIC values with the unfixed occupancy model. Consistent with our intuition about the high detectability of *Polylepis* trees, the fixed occupancy logistic regression received strong support in the model comparison for both *Polylepis* species (delta AIC = 16.48 and 32.89 for *P. sericea* and *P. weberbaueri* respectively), and we base our subsequent inference on these regressions. We identified bird-habitat associations after a visual examination of the biplots vectors with the greatest eigenvalues derived from a Canonical Correspondence Analysis (CCA) (Braak, 1986), as has been done in other *Polylepis* studies (Lloyd, 2008c). We combined bird data from both seasons for each point count, using the encounter rate of each species as measure of their relative abundance for the subsequent CCA analysis. We restricted the analysis to those bird species that were observed in at least 20 independent times over both seasons and over the 130 point count locations. We defined peruvian endemics and threatened bird species according to the IUCN Red List of threatened species (Birdlife International, 2016).

## Results

### Habitat characteristics

Valleys differed significantly in height and composition (e.g., moss, Puna grass, rock) of ground cover, as well as in forest patch sizes and amount of forest within 50 m (Table 1). Interestingly, most structural attributes of the forest, such as DBH, tree height, canopy cover, and tree and shrub density, were similar among valleys.

**Table 1:** Habitat and landscape attributes (Mean ± SD, Min–Max) at five glacial valleys in Huascaran National Park, Peru, 2014–2015. Number of survey points are indicated below valley names. Numbers of trees measured are indicated below tree height and dbh. Statistical differences are in bold *p* < 0.05.

	Total *n* = 130	Llanganuco *n* = 30	Llaca *n* = 20	Ulta *n* = 30	Rajucolta *n* = 20	Parón *n* = 30	Between- localities differences
**Elevation (m)**	**4080 ± 327.8 3302–4678**	**3999 ± 322.6 3468–4513**	**4274 ± 225.1 4007–4610**	**4030 ± 331.9 3515–4495**	**4249 ± 240.4 3965–4678**	**3971 ± 67 3302–4591**	***X***^**2**^**: 14.96,*p*** **< 0.005**
DBH (cm)	29.7 ± 33.84 10–253 *n* = 274	28.9 ± 26.87 10–158 *n* = 69	28.5 ± 22.52 10–117 *n* = 49	23.4 ± 34.86 10–253 *n* = 53	41.2 ± 33.91 10–132 *n* = 33	30.9 ± 43.82 10–219 *n* = 70	*X*^2^: 6.12, *p* = 0.191
Tree height (m)	6.4 ± 2.96 1.5–20 *n* = 274	6.2 ± 2.58 1.5–14 *n* = 69	5.2 ± 2.84 1.8–11 *n* = 49	8.6 ± 3.97 1.6–20 *n* = 53	7.0 ± 2.30 3–16 *n* = 33	5.3 ± 1.72 2–10 *n* = 70	*X*^2^: 1.44, *p* = 0.838
**Ground cover height (cm)**	**0.8 ± 0.34 0.0–1.68**	**1.0 ± 0.35 0.4–1.68**	**0.8 ± 0.22 0.5–1.25**	**0.8 ± 0.42 0.0–1.63**	**0.7 ± 0.19 0.2–1.03**	**0.9 ± 0.27 0.6–1.38**	***X***^**2**^**: 21.17,*p*= 0.001**
Canopy depth (m)	1.4 ± 1.42 0.00–11	1.5 ± 1.12 0.00–4.33	1.3 ± 0.79 0.00–2.63	1.8 ± 2.39 0.00–11	1.00 ± 1.04 0.00–3.5	1.3 ± 0.77 0.00–2.75	*X*^2^: 2.57, *p* = 6.33
Tree density (Ind/100 m^2^)	3.2 ± 3.82 0.00–17.19	4.5 ± 4.96 0.00–17.19	4.0 ± 6.13 0.00–14.01	2.8 ± 3.83 0.00–10.50	2.6 ± 3.57 0.00–5.41	2.4 ± 2.22 0.00–11.78	*X*^2^: 4.70, *p* = 0.320
Bushes density (Ind/100 m^2^)	14.5 ± 12.08 0.00–111.41	24.1 ± 13.18 0.00–111.41	10.2 ± 6.03 2.55–23.87	17.5 ± 14.69 0.32–33.1	9.6 ± 7.63 0.64–46.15	10.5 ± 7.21 2.55–27.37	*X*^2^: 8.59, *p* = 0.072
**Moss cover percent**	**0.3 ± 0.25 0.00–0.94**	**0.3 ± 0.290.00–0.94**	**0.6 ± 0.250.11–0.89**	**0.2 ± 0.21 0.01–0.75**	**0.2 ± 0.18 0.01–0.79**	**0.2 ± 0.15 0.00–0.74**	***X***^**2**^**: 24.48,*p*= 0.001**
**Puna grass cover percent**	**0.3 ± 0.32 0.0–0.95**	**0.4 ± 0.32 0.0–0.95**	**0.2 ± 0.33 0.0–0.91**	**0.4 ± 0.31 0.0–0.95**	**0.3 ± 0.37 0.0–0.94**	**0.1 ± 0.140.0–0.73**	***X***^**2**^**: 29.88,*p*= 0.001**
**Rock cover percent**	**0.4 ± 0.26 0.00–0.95**	**0.3 ± 0.21** 0.00–0.94	**0.7 ± 0.22** 0.29–0.94	**0.3 ± 0.22** 0.02–0.70	**0.3 ± 0.37** 0.02–0.95	**0.3 ± 0.18** 0.03–0.78	***X***^**2**^**: 38.75,*p*= 0.001**
Bare ground cover percent	0.4 ± 0.26 0.00–0.95	0.1 ± 0.18 0.00–0.75	0.0 ± 0.02 0.00–0.06	0.1 ± 0.10 0.00–0.50	0.1 ± 0.06 0.00–0.23	0.1 ± 0.10 0.00–0.36	*X*^2^: 9.95, *p* = 0.041
Canopy cover percent	0.5 ± 0.38 0.00–1.00	0.5 ± 0.40 0.00–1.00	0.6 ± 0.39 0.00–1.00	0.5 ± 0.44 0.00–1.00	0.4 ± 0.34 0.00–1.00	0.5 ± 0.41 0.00–1.00	*X*^2^: 1.12, *p* = 0.892
Slope	23.2 ± 9.89 5–48	18.7 ± 9.17 6–36	25.2 ± 10.80 9–48	23.8 ± 11.00 5–46	24.6 ± 8.30 6–35	25.0 ± 8.9 7–38	*X*^2^: 8.18, *p* = 0.085
**Forest on 50 m-r plot (0.79 ha)**	**0.35 ± 0.26 0–0.79**	**0.39 ± 0.26 0–0.79**	**0.47 ± 0.23 0–0.79**	**0.24 ± 0.25 0–0.79**	**0.27 ± 0.27 0–0.79**	**0.39 ± 0.25 0–0.79**	***X***^**2**^**: 13.53,*p*= 0.009**
**Patch size (ha)**	**31.2 ± 50.68 0–180.48**	**62.4 ± 85.02 0–180.48**	**31.9 ± 29.94 0–61**	**3.9 ± 7.03 0–20.7**	**39.4 ± 43.19 0–85.9**	**21.7 ± 20.65 0–44.3**	***X***^**2**^**: 13.94,*p*= 0.007**
**Distance to the edge (m)**	**−41.5 ± 186.56 −1372–177**	**−7.1 ± 72.79 −315–84**	**31.7 ± 78.83 −165–177**	**−60.2 ± 111.05 −414–60**	**−192.8 ± 403.29 −1372–50**	**−5.2 ± 64.31 −246–67**	***X***^**2**^**: 16.24,*p*= 0.003**

**Figure 2:**
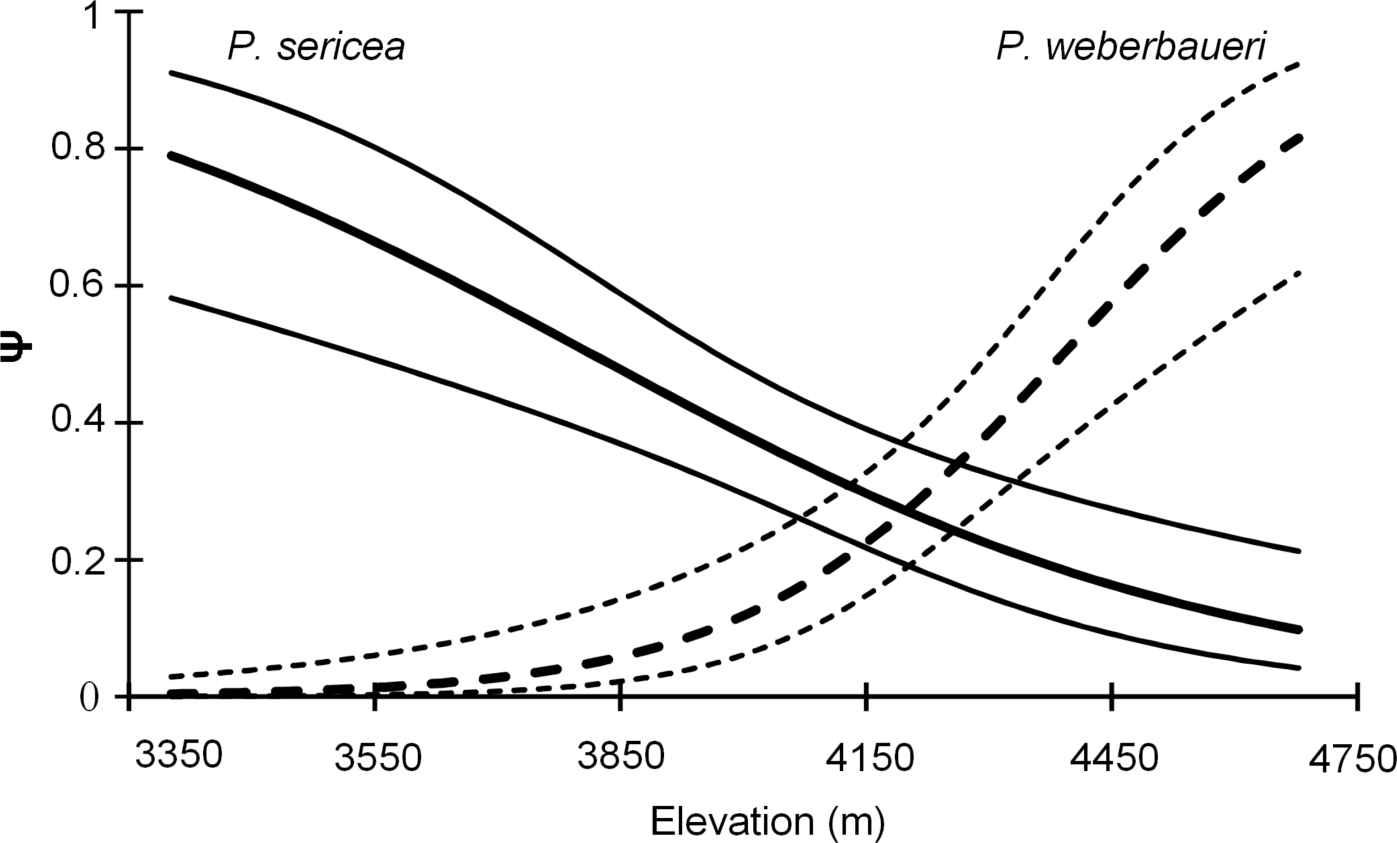
Occupancy estimates *ψ* for *P. sericea* and *P. weberbaueri* along an elevational gradient. Fine lines represent the 95% CI.

Both occupancy models and the NMDS indicated that forest composition changed with elevation (Table 2; Fig. 2; Table S1; Fig. S3). Elevation was a significant predictor of occupancy for the two *Polylepis* tree species within the study area (Likelihood ratio test: *P. sericea*: *x*^2^ = 18.58, *df* = 1, *p* = 0.0001; *P. weberbaueri*: *x*^2^ = 36.98, *df* = 1, *p* = 0.0001) (Table 2); with *P. sericea* being replaced by *P. weberbaueri* as elevation increased. *P. sericea* was the most common in the study area, at 33% occupancy (*Psi*-hat: 0.33; SE: 0.044; 95% CI [0.25–0.43]) compared to 17% for *P. weberbaueri* (*Psi*-hat: 0.17; SE: 0.042; 95% CI [0.10–0.27]). Occupancy probability for *P. sericea* decreased monotonically with increasing elevation, from 0.8 at 3,300 m, 0.5 at 3,870 m and only 0.1 at 4,680 m; whereas *P. weberbaueri* increased more rapidly from 0.1 at 3,980 m, to 0.5 at 4,390 m and to 0.8 at 4,680 (Fig. 2). The two species co-occurred between 4,060 to 4,350 m (Fig. S4).

**Table 2:** Occupancy estimates (Psi) of *Polylepis sericea* and *P. weberbaweri* within five valleys in Huascaran National Park, Peru.

	*Psi*-hat	SE	95% CI	AICc
*P. sericea*				
*p* (1) Psi (Elev)	0.335	0.045	0.25–0.43	154.5
*p* (.) Psi (Elev)	0.335	0.045	0.25–0.43	154.5
*p* (.) Psi (.)	0.354	0.041	0.27–0.44	170.98
*P. weberbaueri*				
*p* (1) Psi (Elev)	0.168	0.043	0.10–0.27	116.52
*p* (.) Psi (Elev)	0.168	0.043	0.10–0.27	116.52
*p* (.) Psi (.)	0.262	0.039	0.19–0.34	149.41

**Notes.**

*p* encounter probability Elev Elevation in m

For other habitat variables, the first NMDS axis was positively associated with elevation, DBH, *P. weberbaueri,* and biomass and negatively associated with groundcover height and shrub density (Table S1; Fig. S3). The second NMDS axis was negatively associated with elevation and positively associated with structural characteristics typical of *P. sericea* forest (e.g., tree height, canopy cover, canopy depth), and landscape characteristics, including amount of forest, patch size and internal distance to the edge. Collectively these axes showed that landscapes at lower elevations had larger patches of forest (∼>10 ha) dominated by *P. sericea,* with smaller and fewer trees and shrubs, resulting in lower biomass overall, than upper elevations. Sampling points at higher elevations, on the other hand, were dominated by smaller patches of *P. weberbaueri* with taller and larger trees, high canopy cover, and comparatively less understory height.

### Habitat and bird community ordination

We recorded in total 8,839 records of 101 bird species at points–with 2,853 observations of 77 species recorded during the dry season compared to 5,986 observations of 88 species in the wet season. Half of these species were difficult to detect, probably due to their secretive behavior and/or their intrinsic low population densities (Lloyd, 2008a), and only fifty bird species were recorded in at least 20 independent detection events and used in the CCA, including 13 species of conservation concern (Table 3) representing 6 Peruvian endemics and 6 IUCN Red List—threatened species and two *Polylepis* specialists (*Grallaria andicolus* and *Xenodacnis parina*).

**Table 3:** Ordination of the 13 bird species of conservation concern for the first four canonical factors from the CCA.

	Axis 1	Axis 2	Axis 3	Axis 4
*Anairetes alpinus*^EN^*ϕ*	**−1.310**	**3.243**	**−2.454**	0.970
*Atlapetes rufigenis*^NTE^	0.208	0.612	**1.003**	**−0.802**
*Cranioleuca baroni*^E^	0.877	**1.054**	0.923	0.256
*Grallaria andicolus*^*ϕ*^	−0.521	0.276	0.518	0.354
*Leptasthenura pileta*^E^	**1.109**	−0.709	0.151	**1.768**
*Leptasthenura yanacensis*^NT^ϕ	**−2.130**	**2.734**	**−2.341**	0.206
*Metallura phoebe*^E^	0.880	−0.499	0.447	0.355
*Oreomanes fraseri*^NT^ϕ	0.332	**1.571**	−0.090	**1.142**
*Microspingus alticola*^ENE^^ϕ^	**1.790**	0.843	0.408	0.279
*Scytalopus affinis*^E^ϕ	−0.525	0.518	0.232	0.777
*Geocerthia serrana*^E^	−0.947	−0.364	−0.455	**−0.999**
*Xenodacnis parina*^ϕ^	−0.327	0.408	0.584	**−0.752**
*Zaratornis stresemanni*^V UE^ϕ	−0.218	**2.608**	**−3.645**	**1.363**

**Notes.**

Higher values are shown in bold.

NT Near Threatened VU Vulnerable EN Endangered (IUCN 3.1 2016) *ϕ* *Polylepis* specialist E Endemic

**Figure 3:**
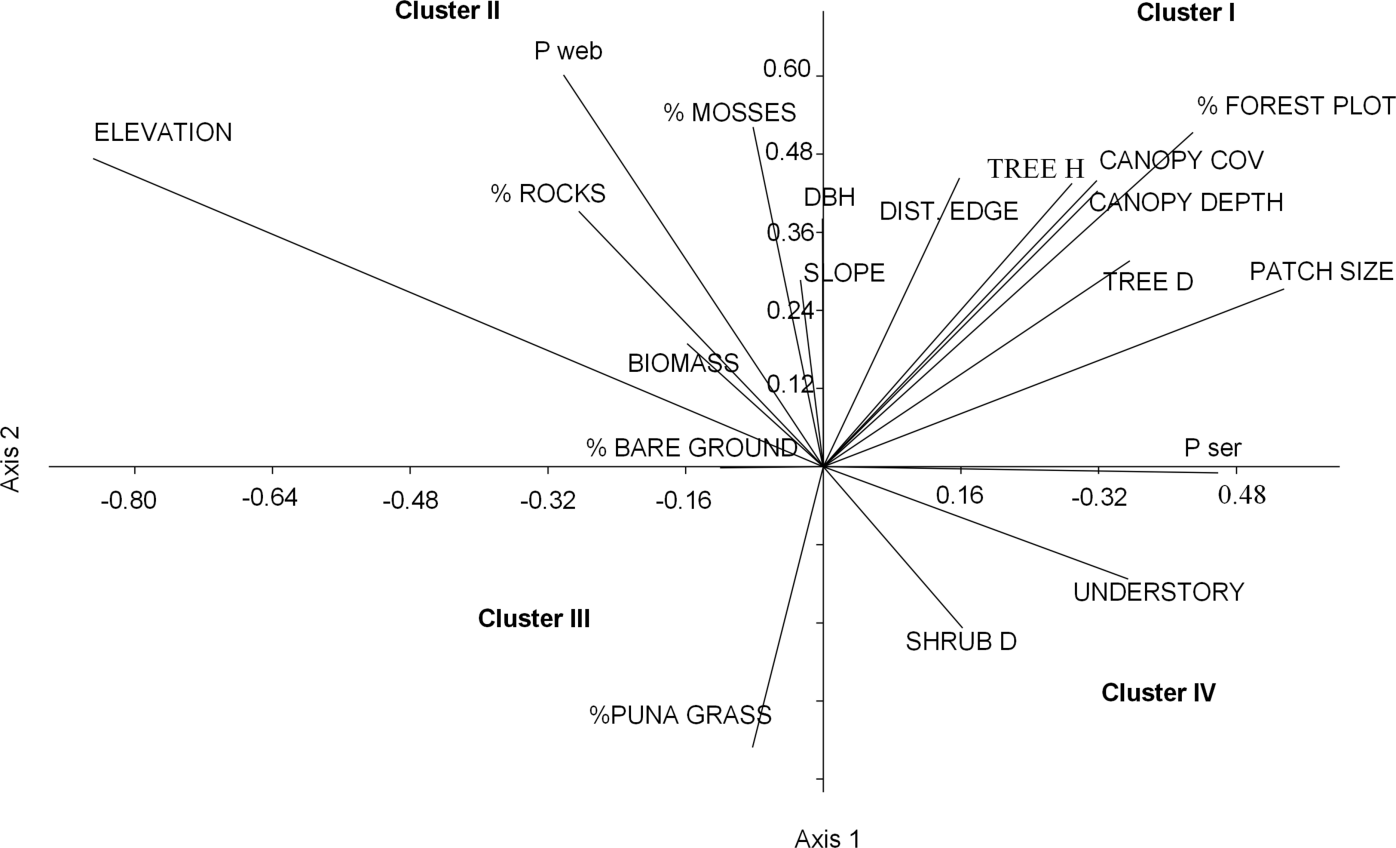
Ordination plot of 19 habitat variables across an elevational gradient of *Polylepis* woodlands along the first two canonical axes from the CANOCO analysis.

Nearly half of the variation in bird communities (48.3%) with respect to habitat and landscape attributes was explained by the first two CCA vectors, with a sum of all eigenvalues of 0.74. The first vector, which explained 28.7% of the variation, was associated with low elevations, large patches of *P. sericea*, high tree densities, dense canopy cover, and tall understory vegetation. The second vector, explaining 19.6% of the variance, was positively associated with small patches of *P. weberbaueri* at high elevation and with tall trees, high canopy cover, abundant mosses, and sparse grass and shrubs (Fig. 3; Table 4).

**Table 4:** Ordination of 19 habitat variables on the first four canonical factors from the CANOCO analysis.

	Factor 1	Factor 2	Factor 3	Factor 4
Patch size ha	**0.536**	0.273	−0.040	0.051
*P. sericea* presence	**0.459**	−0.010	**0.249**	0.184
Forest in 50 m-r plot %	**0.429**	**0.514**	**0.262**	−0.119
Tree density	**0.356**	**0.316**	**0.211**	−0.106
Groundcover height m	**0.354**	−0.172	0.073	−0.138
Canopy depth m	**0.319**	**0.423**	0.137	**−0.217**
Canopy cover	**0.318**	**0.439**	0.156	**−0.216**
Tree height m	0.289	**0.435**	0.109	**−0.200**
Shrub density	0.162	−0.248	0.034	−0.108
Distance to the edge m	0.158	**0.443**	**0.317**	−0.019
DBH cm	−0.001	**0.381**	0.090	−0.022
Slope	−0.026	0.287	−0.125	−0.068
Moss %	−0.081	**0.522**	**0.230**	**−0.208**
Grass %	−0.082	**−0.431**	**−0.242**	**−0.334**
Bare ground %	−0.120	−0.002	**0.228**	0.103
Biomass	−0.158	0.189	0.076	0.022
Rocks %	−0.284	**0.392**	−0.082	−0.036
*P. weberbaueri* presence	−0.302	**0.601**	−0.033	−0.103
Elevation m	**−0.848**	**0.473**	0.095	0.173

**Figure 4:**
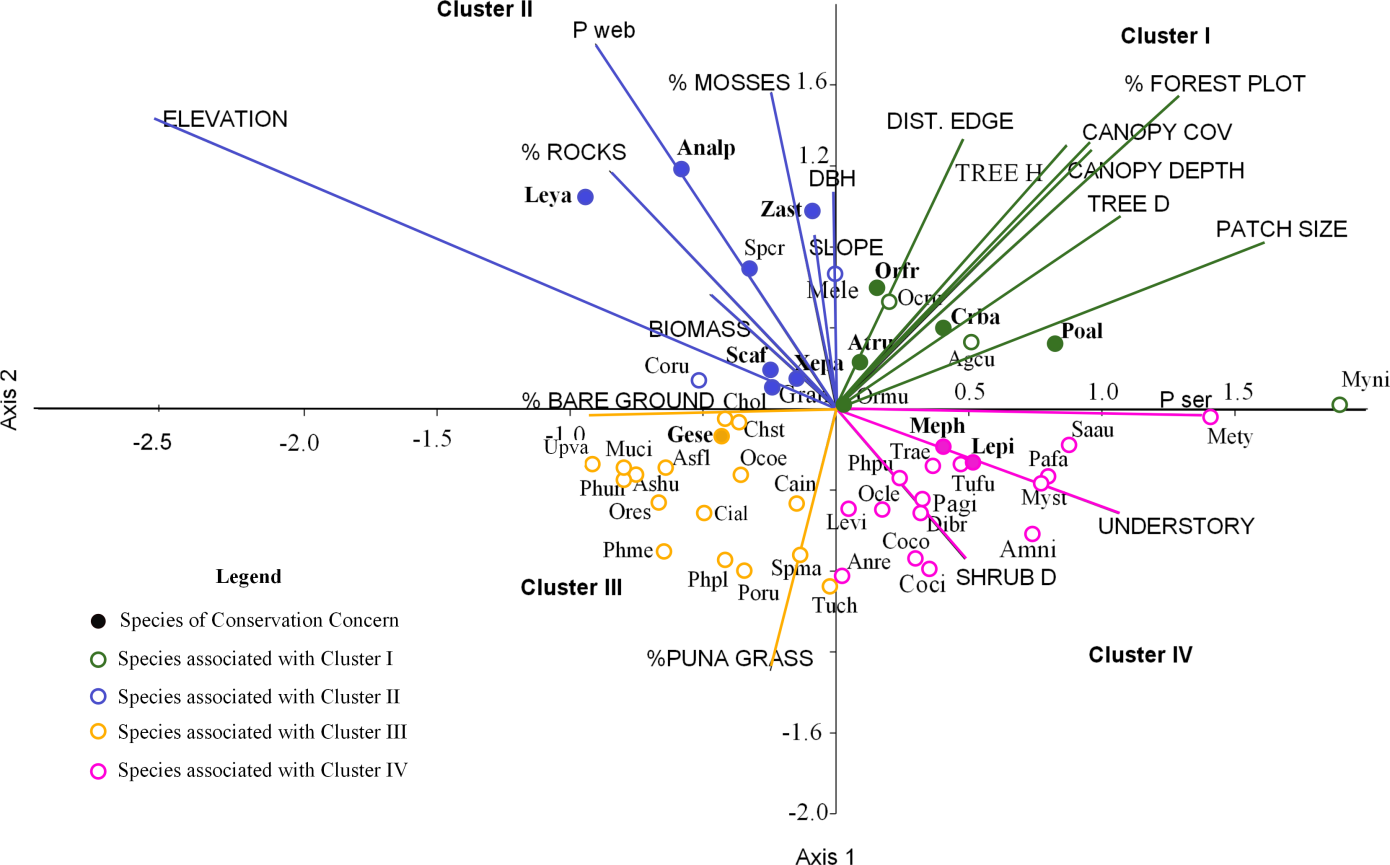
Ordination of 50 bird species points within 19 environmental variables upper case for the first two canonical factors from the CCA *x* and *y* axes. For cluster descriptions: see text. Bird species of concern are in bold type. Agcu, *Aglaeactis cupripennis*; **Analp, *Anairetes alpinus***; Anni, *Anairetes nigrocristatus*; Anre, *Anairetes reguloides*; Asfl, *Asthenes flamulata*; Ashu, *Asthenes humilis*; **Atru, *Atlapetes rufigenis***; Cain, *Catamenia inornata*; Chol, *Chalcostigma olivaceum*; Chst, *Chalcostigma stanleyii*; Cifu, *Cinclodes fuscus*; Coru, *Colaptes rupicula*; Coco, *Colibri coruscans*; Coci, *Conirostrum cinereum*; Crba, *Cranioleuca baroni*; Dibr, *Diglossa brunneiventris*; Gran, *Grallaria andicolus*; **Lepi, *Leptasthenura pileata***; **Leya, *Leptasthenura yanacensis***; Levi, *Lesbia victoridae*; Mele, *Mecocerculus leucophrys*; **Meph, *Metallura phoebe***; Mety, *Metallura tyrianthina*; Muci, *Muscisaxicola cinereua*; Myni, *Myiothlypis nigrocristatus*; Myst, *Myiotheretes striaticollis*; Ocle, *Ochthoeca leucophrys*; Ocoe, *Ochthoeca oenantoides*; Ocru, *Ochthoeca rufipectoralis*; Ormu, *Orochelidon murina*; **Orfr, *Oreomanes fraseri***; Ores, *Oreotrochilus estella*; Pafa, *Patagioenas fasciata*; Pagi, *Patagona gigas*; Phme, *Phalcoboenus megalopterus*; Phpl, *Phrygilus plebejus*; Phpu, *Phrygilus punensis*; Phun, *Prhygilus unicolor*; Poru, *Polioxolmis rufipennis*; **Poal, *Microspingus alticola***; Saau, *Saltator aurantiirostris*; **Scaf, *Scytalopus affinis***; Spcr, *Spinus crassirostris*; Spma, *Spinus magellanicus*; Trae, *Troglodytes aedon*; Tuch, *Turdus chiguanco*; Tufu, *Turdus fuscater*; Geje, *Upucerthia validirostris*; **Gese, *Geocerthia serrana***; Xepa, *Xenodacnis parina*; **Zast, *Zaratornis stresemanni***.

We grouped the habitat associations of birds into four clusters based on their positive or negative loadings on CCA axes 1 and 2 (Fig. 4): (1) habitat structure associated with *P. sericea* dominated forest (e.g., canopy cover, patch size, forest interior (distance to the edge), tree density and height) (Cluster I), (2) habitat structure associated with *P. weberbaueri*, such as higher levels of mosses, rocks, biomass, DBH and slope (Cluster II), (3) grassland associated with Puna or other open habitats (Cluster III), and (4) dense areas with tall herbaceous groundcover and high shrub density (Cluster IV).

Seventeen bird species were strongly associated with *Polylepis* forest. Of these, 9 species were associated with *P. sericea* habitat (Cluster I), including four threatened/endemic species the Rufous-Eared Brush-Finch (*Atlapetes rufigenis*), Plain-tailed Warbling-finch (*Microspingus alticola*), Giant Conebill (*Oreomanes fraseri)* and the Baron’s Spinetail *(Cranioleuca antisiensis baroni*)*;* three widely distributed insectivores: Black-crested Warbler (*Myiothlypis nigrocristata*); Rufous-Breasted Chat-tyrant (*Ochtoeca rufipectoralis*), Brown-Bellied Swallow(*Notiochelidon murina*) and two hummingbirds: Shining Sunbeam (*Aglaeactis cupripennis*) and Tyrian Metaltail (*Metallura tyrianthina*). The other eight species were associated with *P. weberbaueri* habitat (Cluster II). These included the endangered Ash-breasted Tit-tyrant (*Anairetes alpinus*), the near-threatened Tawny Tit-spinetail (*Leptasthenura yanacensis*), the endemic and vulnerable White-cheeked Cotinga (*Zaratornis stresemanni*), the endemic Ancash Tapaculo (*Scytalopus affinis*), and the widespread Stripe-headed Antpitta (*Grallaria andicolus*), White-Throated Tyrannulet (*Mecocerculus leucophrys*), Thick-billed Siskin (*Spinus crassirostris*), and Tit-like Dacnis (*Xenodacnis parina*) (Table 3).

Furthermore, many species were associated with grasslands and shrublands (Cluster III and IV). Seventeen species were associated with grasslands and open habitats (e.g., flycatchers, canasteros, finches, ground-tyrants, earth creepers, and hummingbirds), although only one of these was an endemic species - Striated Earthcreeper (*Geocerthia serrana*). Another sixteen species were associated with shrublands (Cluster IV), including two endemic species—Black Metaltail (*Metallura phoebe*) and Rusty-Crowned Tit-Spinetail (*Leptasthenura pileata*).

## Discussion

*Polylepis* forests in Huascaran National Park and Biosphere support unique bird communities, including several threatened and endemic bird species (Fjeldså & Kessler, 2004; Gareca et al., 2010). Birds generally were associated with four types of habitat within the valleys–(1) lower elevation *P. sericea* forests, (2) higher elevation *P. weberbaueri* forests, (3) grasslands and Puna habitat, and (4) successional shrublands. Although each of these habitats support at least one endemic or declining species, the *Polylepis* forests supports the greatest number of threatened and endemic species. That said, forests varied in terms of their suitability for any given bird species, which were associated with different floristic and structural attributes.

### Floristic shifts in *Polylepis* forests

One unexpected finding of our study was the floristic shift along the elevational gradient, whereby larger patches of *P. sericea* forests (typically below 3,800 m) were gradually replaced by smaller patches of larger and taller *P. weberbaueri* trees at higher elevations (Fig. S3). In some respects, the greater height and larger DBH of *Polylepis* trees at upper elevations, which are harsher environments, is counter-intuitive (Fig. S4). We suggest the pattern stems from lower levels of human activity and resource extraction at higher and more inaccessible areas. Kessler et al. (2014) also found that *Polylepis* trees were marginally smaller in disturbed than undisturbed areas near Cusco, Peru. Thus, high elevation remnants in inaccessible areas may be the only remaining examples of the more “natural” states of *Polylepis* forest, as has been similarly suggested for other Andean plant communities (Sylvester, Sylvester & Kessler, 2014).

An alternate explanation is that floristic shifts reflect differences in the climatic niche optima between species and their different physiological/genetic characteristics. If *P. sericea* and *P. weberbaueri* have different tolerances (e.g., physical, edaphic, climatic and ecological), then the growing number of *Polylepis* reforestation, afforestation and general restoration projects will need to carefully select species. Others have noted that some projects that elect to use other *Polylepis* species (e.g., *P. incana* and *P. racemosa*) are often marked by high mortality of trees after 15–20 years (C Aucca, 2009, pers. comm.). Inappropriate selection of trees might also lead to competition with native species or genetic problems, such as hybridization (Segovia-Salcedo et al., 2011). Several major *Polylepis* restoration projects in Peru and Ecuador report these kinds of problems and are summarized by Segovia-Salcedo et al. (2011), Fuentealba & Sevillano (2016) and LV Morales, 2017, unpublished data. Such attention is also warranted given the widely documented latitudinal variation in structure and floristics of *Polylepis* ecosystems across the Andes (Kessler et al., 2001; Kessler, 2005; Gosling et al., 2009). More broadly, our findings provide an important reminder that any restoration projects must pay careful attention to local and regional context.

### Bird-habitat associations along the elevational gradient

Bird communities changed markedly along elevational gradients in response to shifts in habitat and floristic structure within each valley. At lower elevations (3,300 m–4,000 m); four birds of concern (Giant Conebill (*Oreomanes fraseri*), Plain-tailed Warbling-finch (*Microspingus alticola*), Baron’s Spinetail (*Cranioleuca baroni*), and Rufous-eared Brush-finch (*Atlapetes rufigenis*)) and three other widely distributed species (the Rufous-breasted Chat-tyrant (*Ochthoeca rufipectoralis*), the Black-crested Warbler (*Myiothlypis nigrocristata*) and the Shining Sunbeam (*Aglaeactis cupripennis*)) were strongly associated with large patches of mature *P. sericea* forests.

Of these species, the near-threatened Giant Conebill (*Oreomanes fraseri*) is already known to be a specialist that nests (Cahill, Matthysen & Huanca, 2008) and forages on *Polylepis* bark (Fjeldså & Krabbe, 1990; Servat, 2006; Lloyd, 2008b). We also found that Giant Conebills were associated with large diameter trees and the interior of forest patches, which is consistent with previous studies that show that the Giant Conebill favored large trees in mature forests (Lloyd, 2008a) and avoided edges (Cahill & Matthysen, 2007).

The other three species are recognized as endemic and threatened species but otherwise their ecology is poorly known (Huffstater, 2012; Schulenberg & Jaramillo, 2015). Both, the Plain-tailed Warbling-finch and the Baron’s Spinetail were associated with the interior of large patches of mature *Polylepis* forest at relatively much lower elevations than the Giant Conebill, whereas the Rufous-eared Brush-finch was the most tolerant of small patches and near edges. Interestingly, the Plain-tailed Warbling-Finch, which is listed as a rare and endangered (EN) by Birdlife International, was relatively common at lower elevations in our study area and was often seen foraging in pairs, familiar groups and/or mixed flocks in *Polylepis sericea* mixed forest with *Gynoxys* and *Alnus*. Further population studies are needed to better understand its status and specifically the extent to which observations reflect new distributional information or local population changes. Three other species widely distributed along the Andes, the Rufous-breasted Chat-tyrant (*Ochthoeca rufipectoralis*), the Black-crested Warbler (*Myiothlypis nigrocristata*) and the Shining Sunbeam (*Aglaeactis cupripennis*) were associated with the interior of *P. sericea* forest and only occurred at lower elevations. At higher elevations (>4,000 m), some of the most specialized and endangered species were associated with *P. weberbaueri* forests of any size that were less disturbed, likely due to inaccessibility in terms of location and terrain (e.g., steep and rocky terrain. Surprising, though, these endangered birds were not associated with the patch size forest. Among these was one of most highly threatened species in the Andes, the Ash-breasted Tit-tyrant (*Anairetes alpinus*)) (EN), which has a global population estimated in 780 individuals that declined by 10–19% between 2002 and 2012 in Peru and Bolivia (US Fish Wildlife Service, 2012). Two other *Polylepis* specialist of concern, the endemic and vulnerable White-cheeked Cotinga (*Zaratornis stresemanii)* and the near threatened Tawny Tit-spinetail (*Leptasthenura yanacensis*), were strongly associated with mosses and rocks within remote *P. weberbaueri* stands, irrespective of patch size. Previous studies have also reported that these species are abundant in small patches of *Polylepis* at high elevations (Lloyd, 2008c; Lloyd, 2008b; Sevillano-Ríos, Lloyd & Valdés-Velásquez, 2011), though the sensitivity of the Tawny Tit-spinetail to edges in Bolivia is known to be highly variable (Cahill & Matthysen, 2007). Our observations suggest that individuals were not restricted to any single small patch, and instead patrolled multiple patches within the landscape, raising the possibility that they are adapted to naturally fragmented landscapes.

These patterns carry three key conservation implications. First, at high elevations, conservation of forest patches, regardless of size, is likely essential to maintaining populations of specialized and threatened species. However, we recognize that the substantial correlation observed among *P. sericea*, patch size and low elevations makes difficult a better interpretation of the role of small patches at lower elevations. However, we recognize that the strong correlations among *P. sericea,* patch size and low elevations prevents us from fully understanding the importance of small patches at lower elevations. Likewise, there remains the possibility that a species was simply area-sensitive, specializing on large patches, rather than actively preferring or specializing on *P. sericea* or low elevation habitats. Secondly, because previous bird surveys within *Polylepis* forest of the Cordillera Blanca occurred at lower elevation (i.e., below 4,300 m), abundance of certain species, such as Ash-breasted Tit-tyrant, may have been substantially underestimated (Fjeldså, 1987; Frimer & Nielsen, 1989; Servat, 2006; Sevillano-Ríos, Lloyd & Valdés-Velásquez, 2011). Lastly, elevational changes in bird communities show that conservation efforts must occur throughout the elevational gradient rather than focusing only on particular habitat or landscape attributes (e.g., patch size).

### Vulnerability to climate change

Although most of our understanding of how birds are or will be affected by climate change comes from studies in temperate zones (Crick, 2004; Wormworth & Mallon, 2007, but see Forero-Medina et al., 2011; Şekercioğlu, Primack & Wormworth, 2012), there is widespread agreement about the high vulnerability of tropical montane bird communities (Herzog et al., 2011; Wormworth & Sekercioglu, 2011; Şekercioğlu, Primack & Wormworth (2012)). Andean studies showed that since 1970, Andean glaciers have been reduced by 20–30% (Vuille et al., 2008), at a rate of 3% per year (Fraser, 2012; Rabatel et al., 2013), and the climate has become hotter and dryer as a whole (Baraer et al., 2012; Georges, 2004; Vuille et al., 2008; Mark et al., 2010), where water stress, in particular, is expected to become more acute in the future (Mark et al., 2010). Given that the most severe contractions of *Polylepis* forests occurred in dry and warm periods (Gosling et al., 2009), for the changing climate is likely to profoundly affect *Polylepis* ecosystems and associated bird communities. Moreover, recent studies suggest that many species—especially endemics and threatened species—have limited potential to adjust their requirements to new climatic conditions, especially in cases where their niches are thermally constrained (Malcolm et al., 2006; Forero-Medina et al., 2011; Şekercioğlu, Primack & Wormworth, 2012; Hannah, 2015). For some of the most vulnerable species, upslope elevational shifts might alter species interactions and increase extinction risk for less competitive and more specialized species (Brommer & Møller, 2010; Brotons & Jiguet, 2010). In consequence, the low population densities (Lloyd, 2008c; Sevillano-Ríos, 2016), and high degree of endemism (Lloyd, 2008a), and small distribution ranges (Schulenberg et al., 2010) make *Polylepis* birds vulnerable to stochastic events, which can extinguish entire populations rapidly (Şekercioğlu et al., 2008).

### Implications for conservation

Our research has three main implications for conservation: (1) large patches (>10 ha) of mature *Polylepis* at low elevations should be a cornerstone for Andean bird conservation given their ability to support diverse bird communities, including several endemic and specialist species; (2) small *Polylepis* patches (<2 ha) at high elevations provide unique habitat to severely threatened species and, thus, are critical refuges that also warrant protection; and (3) habitats that are usually less recognized for their conservation value, such as grasslands and shrublands, support surprisingly large numbers of species and even several endemics. However, the degree to which the suitability of grassland and shrubland habitats is related to proximity of *Polylepis* forest for roosting warrants further study. An important caveat of our findings is that we focused on habitat use and associations and, therefore, cannot speak to the quality of the habitat nor the extent to which it affected condition, reproduction, or survival. Given the paucity of demographic information on birds using *Polylepis* forest, additional work is required to evaluate habitat quality and identify the key features required to support populations.

Although we have emphasized the value of forests, other native habitats associated with the *Polylepis* ecosystem play important roles sustaining regional bird diversity. In particular, we note the diverse birds associated with Puna grasslands and shrublands. Although these environments are often considered hostile for some birds (Lloyd & Marsden, 2008), they were heavily used by several hummingbirds, including the endemic *Metallura phoebe*, tyrants, flycatchers, canasteros and finches. These matrix habitats may be important in maintaining connectivity among *Polylepis* patches (Fjeldså & Krabbe, 1990; Kessler, 2006; Tinoco et al., 2013), especially when comprised of *Gynoxys,* a common woody plant known to host abundant and diverse arthropods. At the same time, due to the fact that these areas usually are massive compared to *Polylepis* forest, they may support a high number of species, although the value of open habitats may depend, in part, on the proximity to *Polylepis* forest, as we observed grassland/shrubland birds roosting in forests at night. Thus, *Polylepis* forest may play complementary roles supporting the broader bird community in the valleys.

Our study highlights the importance of in-depth research within globally threatened ecosystems to informing conservation and decision-making. Sometimes, it is easy to assume that unique or rare ecosystems possess relatively little within-system variation, but we show that even subtle differences in floristics and structure may strongly influence bird communities. Further studies on these types of Andean ecosystems, including the Yungas, Paramos and wetlands, would contribute to our understanding of how environmental differences within each ecosystem drives the abundance of many other bird species of conservation concern. However, although this type of study helps to understand the ecological relationships of globally threatened ecosystems and is of great importance for both conservation and environmental decision-making over tropical regions, the number of studies are still reduced compared with the temperate zones. During the last International Congress of Ecology and Conservation of *Polylepis* forests in Jujuy, Argentina, the need to carry out and publish these types of studies (LV Morales, 2017, unpublished data). Most studies come only from few areas, which makes difficult to generalize ecological patterns and use them to improve wider conservation efforts (D Renison, 2007, unpublished data). Further studies over unstudied *Polylepis* areas and other threatened ecosystems will be important to improve the conservation efforts along tropical zones.

##  Supplemental Information

10.7717/peerj.3220/supp-1 Supplemental Information 1 Sevillano, 2016, *Polylepis* birds data base.

10.7717/peerj.3220/supp-2 Table S1 Axis scores based on non-metric multidimensional scaling NMMS based on 19 habitat variables Score values greater than 0.5 are in bold.

10.7717/peerj.3220/supp-3 Figure S1 Location of study sites along the elevation gradient in *Polylepis* forest of Huascaran National Park, Peru Five glacial valleys, Paron, Llanganuco, Ulta, Llaca and Rajucolta were surveyed for birds and habitat characteristics in 130 points from 3,300 to 4,700 m.

10.7717/peerj.3220/supp-4 Figure S2 Variogram with respect to species richness for the dry (A) and wet (B) season Sill: 0.08 & 0.021, range:0, nugget:0.05 & 0.02 for each season respectively.

10.7717/peerj.3220/supp-5 Fiure S3 Non-Metric Multidimensional Scaling NMMS ordinations of 130 points based on 19 habitat and physical characteristics along a 2-dimensional axis coordinates For visualization purposes, dot points represent locations below 3,800 m, cross points between 3,800 to 4,200 m and square points over 4,200 m in elevation.

10.7717/peerj.3220/supp-6 Figure S4 Tree height and DBH circle size of *Polylepis sericea* (red) and *P. weberbaueri* (black) trees measured at 130 points along an elevational gradient in five valleys in Huascaran National Park, Peru

## Additional Information and Declarations

### Field Study Permissions

The following information was supplied relating to field study approvals (i.e., approving body and any reference numbers): Field study was approved by Servicio Nacional de Areas Naturales Protegidas del Peru (SERNANP) (Resolucion N. 014-2014).

### Data Availability

The following information was supplied regarding data availability: Sevillano Rios, Cristian Steven (2017): Avian community structure and habitat use of Polylepis forests along an elevation gradient DataBase. Figshare. https://doi.org/10.6084/m9.figshare.4123434.v1 Retrieved: 21 09, Apr 21, 2017 (GMT)
