# Supplementary material for: Avian community structure and habitat use of Polylepis forests along an elevation gradient
Source: PeerJ. 2017 Apr 27;5:e3220. doi: 10.7717/peerj.3220 (PMC5410164; doi:10.7717/peerj.3220)
Supplement: Table S1 — Score values greater than 0.5 are in bold. [file peerj-05-3220-s002.docx]

**Table S1.**

| **Habitat variables** | **Axis 1** | **Axis 2** |
| --- | --- | --- |
| Elevation m | **0.613** | **-0.504** |
| Slope | 0.067 | -0.056 |
| Percent Mosses | 0.226 | 0.174 |
| Percent Grass | -0.236 | -0.220 |
| Percent Rocks | 0.144 | -0.029 |
| Percent Bare ground | 0.042 | 0.007 |
| DBH cm | **0.717** | 0.352 |
| Tree height m | 0.282 | 0.477 |
| Groundcover height cm | -0.326 | 0.134 |
| *P. sericea* presence | -0.067 | 0.439 |
| *P. weberbaueri* presence | 0.475 | -0.103 |
| Tree density D. per 100 m^2^ | 0.037 | 0.361 |
| Shrub density D. per 100 m^2^ | -0.240 | 0.098 |
| Canopy covers | 0.144 | **0.504** |
| Canopy width m | 0.182 | 0.450 |
| Biomass | **0.834** | -0.010 |
| Percent Forest on 50 m-r plot | 0.075 | **0.528** |
| Patch size ha | -0.162 | **0.521** |
| Distance to the edge m | 0.187 | **0.553** |
