# Supplementary figures and images for: Avian community structure and habitat use of Polylepis forests along an elevation gradient
Source: PeerJ. 2017 Apr 27;5:e3220. doi: 10.7717/peerj.3220 (PMC5410164; doi:10.7717/peerj.3220)

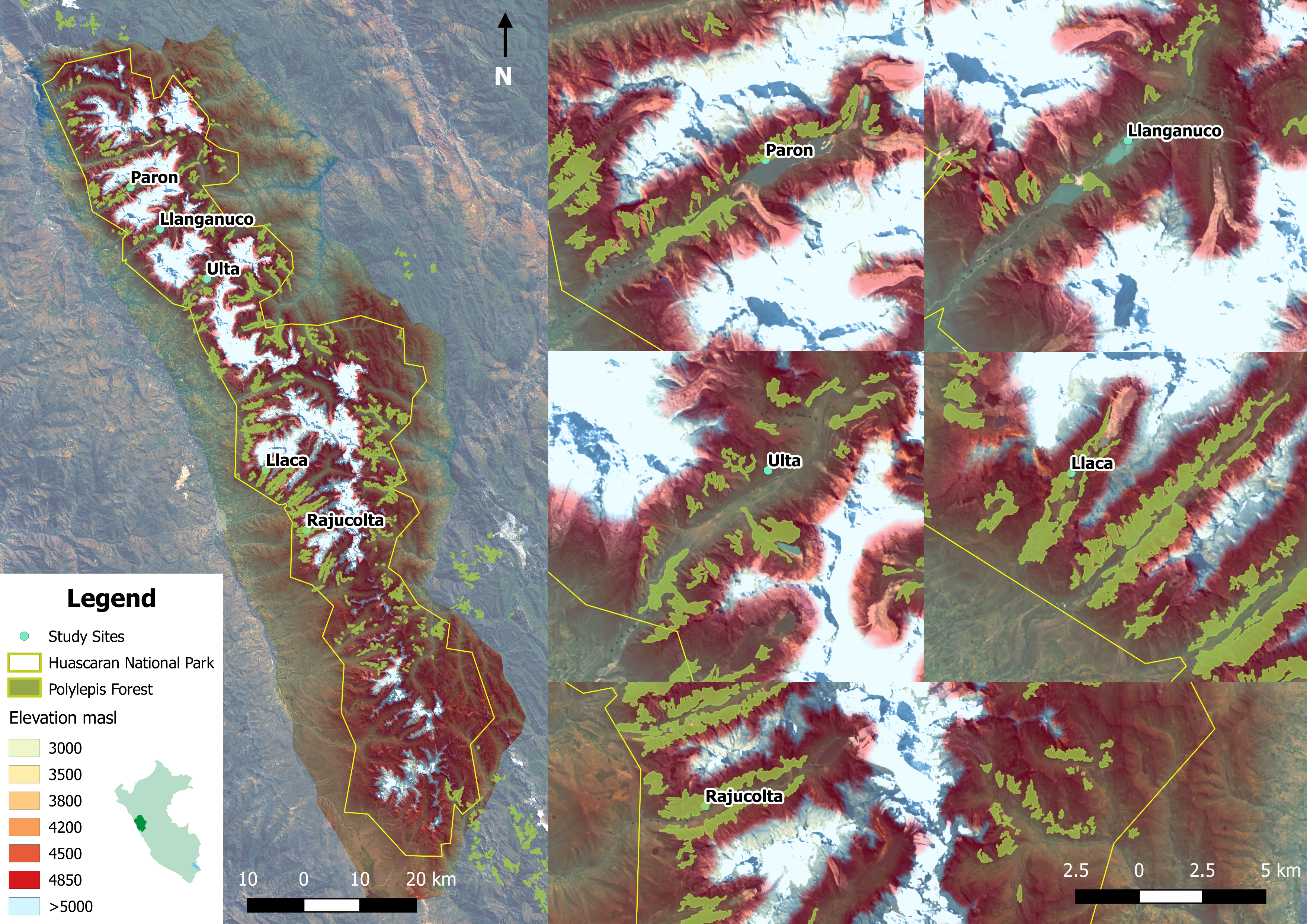

Supplement: Figure S1 — Five glacial valleys, Paron, Llanganuco, Ulta, Llaca and Rajucolta were surveyed for birds and habitat characteristics in 130 points from 3,300 to 4,700 m. [file peerj-05-3220-s003.png]

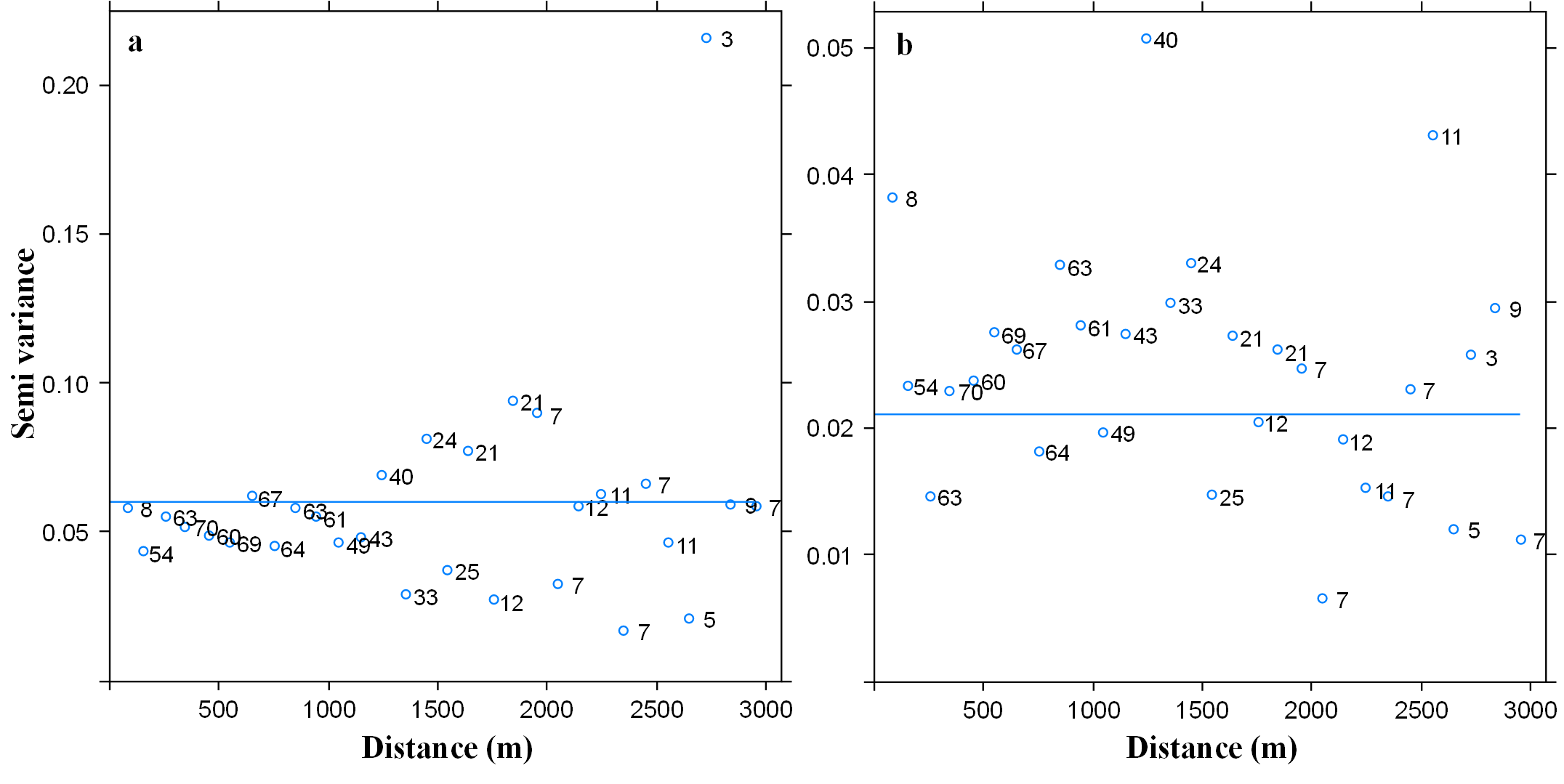

Supplement: Figure S2 — Sill: 0.08 & 0.021, range:0, nugget:0.05 & 0.02 for each season respectively. [file peerj-05-3220-s004.png]

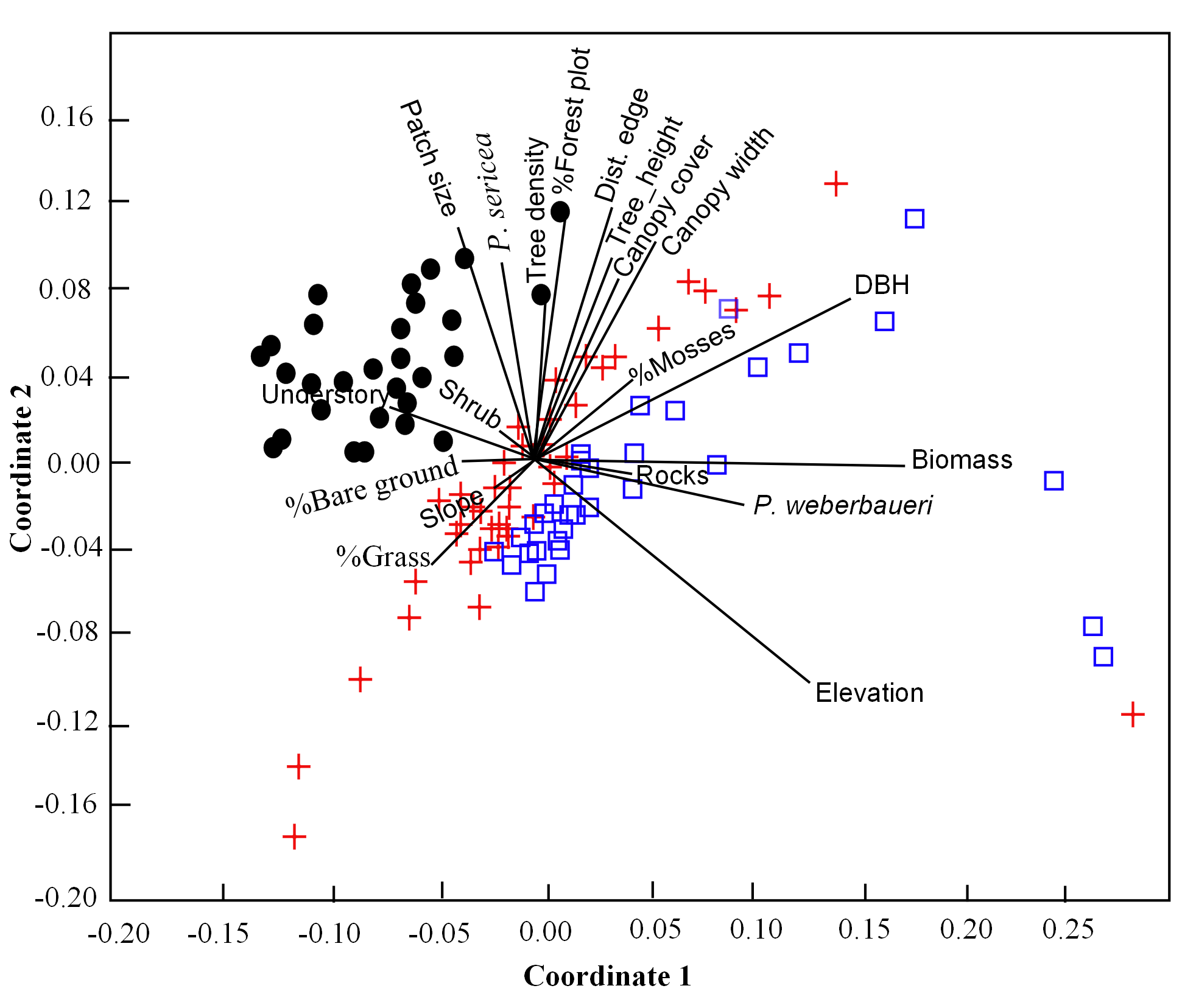

Supplement: Fiure S3 — For visualization purposes, dot points represent locations below 3,800 m, cross points between 3,800 to 4,200 m and square points over 4,200 m in elevation. [file peerj-05-3220-s005.png]

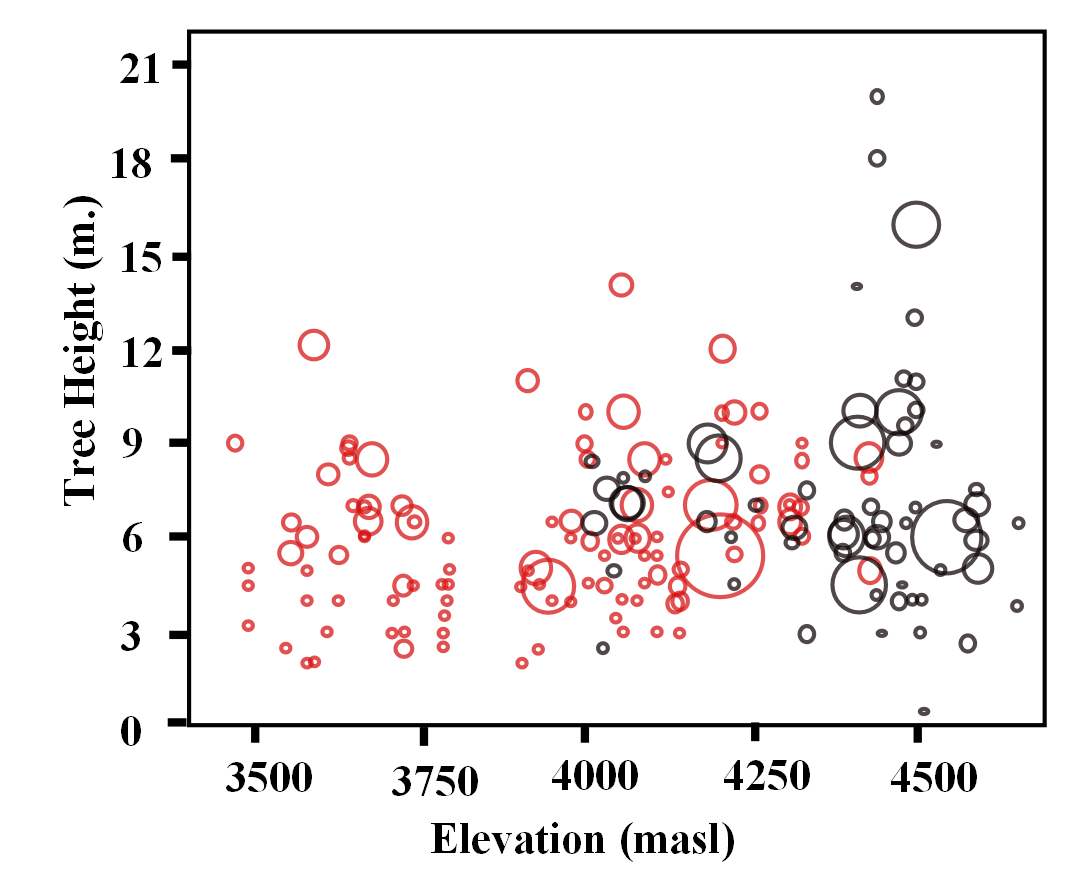

Supplement: Figure S4 [file peerj-05-3220-s006.png]
